# Counter-Prescribing

**Published:** 1888-08

**Authors:** 


### COUNTER-PRESCRIBING.

At a recent meeting of the Atlanta Society of Medicine, one of the members offered a resolution pledging the members to boycott all drug-stores where this pernicious practice was indulged in. The resolution was not passed because the members believed the boycott would be universal, and therefore impracticable.

The subject of counter-prescribing is like the sea serpent: it rises to the surface periodically, is vigorously discussed, but nothing is ever done about it. We fear that a *practical* remedy against this evil will always prove as elusive as the mythical reptile referred to.

### ITEMS.

DR. MARK HOVELL threatens to sue the London *Times* for publishing assertions with reference to his action in the case of the late Emperor of Germany, which are regarded by Dr. Hovell as libelous.

HONOR TO AN ENGLISH OVARIOTOMIST.—At the commencement exercises of Union College, June, 1888, the honorary degree of L.L. D. was conferred upon Mr. Lawson Tait, F. R. C S. Prof. Gynecology, Queen's College, Birmingham, Eng.

THE NEW YORK POLYCLINIC.—The Faculty of the New York Polyclinic have decided to increase the clinical facilities of this institution by establishing a spacious hospital immediately connected with the college building. It will be opened for the reception of patients in October next.

IT is announced that the University of Pennsylvania will soon begin the construction of a maternity ward on the grounds of the University Hospital. The building is to consist of a central portion and two wings. Sufficient money is already on hand to complete the erection of one wing.

DR. BEN. BIZZELL, brother of Dr. W. D. Bizzell, of our city, first-honor man at the Southern, 1887, received his ad-eundem from the College of Physicians and Surgeons, New York, May 12th. After a rigid competitive examination, he has been appointed Medical Inspector of the port of New York. Good for Ben.

DIET-TABLES.—Messrs. Reed & Carnrick, of New York, have issued some very neat, useful and time-saving diet-tables for the convenience of physicians. They are designed to be given to patients or nurses, and will not only save much time now spent in giving directions and answering questions, but will secure greater accuracy. They relate to a great variety of diseases and diseased conditions. Sent free on application.

AT the recent meeting of the Pennsylvania State Medical Society, Dr. Wood, of Pittsburg, amused the Society by moving the adoption of a resolution that the President-elect shall take the following "hypocritic oath;" "Having been duly elected President of this Society, do you promise to hold the Pennsylvania Medical Society, as it has been held by many illustrious men, as a stepping-stone to success, as a round in the ladder of fame, as a lemon to be squeezed, as a lever to raise your hopes, as a block and tackle to exalt your ambition, as a peacock's feather in a jackdaw's tail, as a lion's skin on a sheep, a spur on knighthood's heel, a garter on the leg of a courtier, a medal on the breast of a hero and a convenient method of advertising your business, and that as soon as your time expires you will forever turn your back on it and ignore it? Selah!"

A GREAT CAMPAIGN OFFER.—The Weekly Courier-Journal has now the largest circulation of any Democratic newspaper in the United States, and its publishers, to further extend its circulation, offer to send it, postage prepaid, from June 4th, 1888, to December 31, 1888—thirty-one issues—for only fifty cents.

Subscriptions sent before June 4th will be entered from that time, but those received after June 4th will be entered from the date received, to expire December 31, 1888.

The rate to clubs of eight and over is extremely liberal. A sample copy of the Weekly Courier-Journal, containing its great campaign offer, can be procured, free of charge, by addressing W. N. Haldeman, President Courier-Journal Company, Louisville, Ky.

The subscription price of the Daily Courier-Journal, without the Sunday issue, is ten dollars ($10) a year. Price of Sunday Courier-Journal is two dollars ($2) a year.

CHEAP PHYSIC.—The following is clipped from a Tennessee paper. Comment is unnecessary:

Medical Partnership.—Drs. A. C. Blevins and J. F. Abel have formed a partnership for their mutual benefit, as well as for the benefit of the general public. In the future they will practice medicine and surgery in Dayton and Morgantown and vicinity. Whenever it is desired, the firm will engage to give their medical attention to families for the very reasonable sum of 75 cents per month, and to single men at 40 cents per month, payable monthly in advance. This mode of medical practice has been in vogue in many large cities for some considerable time, and it has in the majority of cases worked well. It is a great saving to everybody; but especially so to workingmen, who are heads of families. Workingmen can save money every time. We have seen the system tried and it works well.

P. S.—Obstetrical and venereal practice is not included in the above terms of agreement.

AT the last meeting of the Ophthalmological and Otological Section of the New York Academy of Medicine, the following motion was made and carried :

"That a committee be appointed, of which the chairman of the section, Dr. David Webster, be a member, whose duty it shall be to obtain a good photograph of the late Dr. Cornelius R. Agnew, for the purpose of having engravings suitable for framing made from this. The right of issue and sale of such engravings shall be given to some first-class publisher, if practicable ; if not, the committee shall offer them to the profession at cost."

In accordance with the above, a committee has been appointed. Members of the profession, who desire such an engraving, accompanied with an autograph signature, should send their names and addresses to the Secretary of the Committee, Dr. Charles H. May, 640 Madison Avenue, New York city, at once. When all such names shall have been recorded, those who have requested a copy of the engraving will be notified of the cost of the same, either by the publisher, or by the committee having the matter in charge.

AN ARKANSAS ECLECTIC'S DISCOVERY.—When I first began the practice of medicine it seemed to me that every female patient I had needed pulsatilla and macrotis, and I accordingly, for a great while, gave them; frequently, however, I would give pulsatilla alone; and the strangest thing was that within twelve months every patient that had taken pulsatilla gave birth to a child, and one or two of them misses—suspicious characters who had doubtless been gratifying their passions for sometime, which had never proved itself.

I have since been studying the action of pulsatilla in this direction, and you need not doubt that it will cause conception in a great many cases. So, if you meet with a married couple who are hunting a child to adopt, give them pulsatilla and send them home. I know it will not cause conception without coition, but I am sure that it exerts a wonderful influence with those who are cohabiting; that if one will put a few drachms in a public well, every woman in town who is cohabiting will bring a child. So I would warn the ladies who are guilty of this practice, and desire to keep it to themselves, to beware of pulsatilla.—*Eclectic Medical Journal*.

THE MEDICO-CHIRUGICAL COLLEGE, of Philadelphia, and the Philadelphia Dental College have placed a contract for a large building to accommodate both schools.

DRUG STORES IN GERMANY.—A correspondent, writing from Dresden, says: "The drug stores have a curious way here of shutting up just about the time you want them. And as soon as it begins to grow dark, down go the shutters; and if you need anything, you go to a little bell-handle outside of one of the iron shutters and ring it. Then you hear some one at a crank inside; the massive frame rolls up and a head looks out of the window. Finally the man or boy inside opens part of the window, and you talk through a pane of glass and make known your wants. Instead of getting angry at being aroused, the man begs your pardon for keeping you outside and says: 'I thank you for your order.' If you have not the exact change, and the man inside is in the same predicament, he will beg you most politely, and thank you, to allow him to change it. Having done so, he will thank you for calling (evidently taking the visit as a social one), bow, close his little peep-hole, bow again, and then smile sweetly as he grinds down his iron shutter and his smiling face is lost to view. How different from the druggists in America! I remember I once woke one up in the States, and he came downstairs with a shot-gun after me. But, as I remarked before, they have a curious way of doing things in Dresden."—*Philadelphia Ledger*, June 12, 1888.

DR. H. V. M. MILLER, one of the editors of this journal, with his wife, left two weeks ago for Waynesville, North Carolina, where they will spend the summer. That he will enjoy his sojourn in the mountain, surrounded by magnificent scenery and refreshing breezes, and that his associates will delight to listen to his sparkling wit and learned discussions, it is unnecessary to say. It is the earnest wish of his many friends that he may return invigorated by his trip.

DR. JOSEPH P. LOGAN and wife leave to-day for an extended tour through the Northwest and West. They go direct to New Mexico, Salt Lake City and extend their trip as far as California. They will be absent a number of weeks. Dr. Logan is an ex-editor of this journal and one of the most distinguished physicians in the State.

THE Government Health Reports state that Plant City, Florida, has again become infected with yellow fever. There were two deaths in June. Active measures have been taken to isolate the town.

THE SOUTHERN SURGICAL AND GYNECOLOGICAL ASSOCIATION will meet in Birmingham on the 11th of September and continue in session three days.

The following are some of the papers which will be presented, viz.: "Floating Kidney with Vicarious Menstruation," by Dr. DeSaussure Ford, of Augusta, Ga.; "The Medical Treatment of Fibrous Tumors of the Uterus," by Dr. Bedford Brown, of Alexandria, Va.; "Indications for Operative Interference in Cerebral Troubles," by Dr. T. O. Summers, of Jacksonville, Fla.; "Super-involution of the Uterus following Trachelorraphy," by Dr. Virgil O. Hardon, of Atlanta, Ga.; "Electrolysis in the Treatment of Urethral Strictures," by Dr. S. M. Hogan, of Union Springs, Ala.; I. "Dermoid Cysts of the Coccygeal Region" "II. "Electrolysis in Gynecology and Surgery," by Dr. E. J. Beall, of Fort Worth, Tex.; "Interesting Cases in Surgery," by Dr. R. M. Cunningham, of Pratt Mines, Ala.; "Antiseptics in Surgery and Gynecology," by Dr. F. T. Merriwether, of Asheville, N. C.; "Gastrostomy," by Dr. W. B. Rogers, of Memphis, Tenn., "The Treatment of Fractures with Plaster of Paris," by W. F. Westmoreland, Jr., Atlanta, Ga.; and papers by Drs. W. Locke Chew, M. C. Baldridge, W. T. Briggs, Richard Douglas, Fred. W. Parham, E. P. Sale, Jno. Herbert Claiborne, Duncan Eve, J. W. Long, Jno. A. Page and others.

The President's address, by Dr. W. D. Haggard, will be delivered at 11 A. M. of the 1st day, and the annual oration, by Dr. W. F. Hyer, at 8 o'clock P. M. of the same day at O'Brien's Opera House.

The Mendelssohn Club will give a complimentary concert on the evening of the oration. Excursions will be had to Lake View, East Lake, North Birmingham, Easley City and Bessemer for the benefit of the members, that they may see Birmingham and its beautiful surroundings. A banquet will be tendered the visitors and members of the association on the evening of the 2d day.

Reduced rates have been secured over all railroads, viz., one full fare going and ⅓ on return. The profession are invited to be present.

LIABILITY OF DRUGGISTS POR CLERKS' MISTAKES.—The Supreme Court of Ohio has recently reiterated the general rule of the liability of druggists for negligence in putting up medicines. In this case the drug clerk, when asked for "oil of sweet almonds," carelessly gave the "oil of bitter almonds," and the plaintiff's wife died almost immediately after taking the poison. There was nothing on the bottle to indicate that it was a virulent poison, and it was clear in the evidence that there was gross negligence on the part of the clerk. The druggist denied his personal liability for his clerk's mistake, but at the trial the court decided against him, and the Supreme Court affirmed the decision. This ruling is fully in accord with that of the courts of other States, and probably no tribunal would relieve a druggist under similar circumstances.—*Med. Times.*

"THE MAN WITHOUT A LARYNX," who has been an object of curiosity to the Paris doctors for sometime, recently died in attempting to remove, cleanse and replace the canula in his throat himself, instead of going to the Saint Louis Hospital, as he had always done before.

